# Clinical Examination of the Urine

**Published:** 1853-06

**Authors:** John Locke

**Affiliations:** The President, Professor of Chemistry and Pharmacy, in the Medical College of Ohio, and Published by request of the Society


### SELECTIONS.

*Clinical Examination of the Urine.* A paper read before the Cincinnati Medical Society, Feb. 1st. 1853. By the President, JOHN LOCKE, M.D., Professor of Chemistry and Pharmacy, in the Medical College of Ohio, and Published by request of the Society.

(CONTINUED FROM PAGE 66.)

THUS procured, nitrate of urea is a beautiful article, almost white, and with so high a pearly lustre, as to approach a silver bronze. The crystals are in minute scales, and when pressed in a thin layer upon paper, exhibit a softened or selenite metallic lustre. If the *quantity* of the urea be sought after, the nitrate should be dried in a moderately warm place, and weighed; allowing 48 parts of pure urea, to every hundred parts of the nitrate.† But no attempt should be made to try the nitrate by a heat stronger than can be borne by the hand, without pain If it should be desirable to procure the pure urea, add carbonate of potassa to the nitrate, and decompose it, forming nitrate of potassa, and freeing the urea, which may be dissolved out by hot alcohol. The alcohol being evaporated, the urea will remain in lustrous silky crystals.

Mr. Alexander, now an M.D. of the Medical College of Ohio, then a pupil of Prof. Lawson, after receiving instructions in the manipulations, succeeded in procuring several fine specimen results in the analysis of urine. Amongst other things, he showed me some superior crystals of nitrate of urea, one of which, under the microscope, was so instructive in reference to the crystalization of that substance, that I immediately made a sketch of it. The outlines of this crystal are all parallel to the sides of an equilateral triangle. The crystal itself, is an irregular hexagon, but with every angle either 60° or 120°, and although the lines which formed the figure, are 14 in number, they have only three directions.

9*th. Process for Uric Acid.*—The quantity of uric acid in human urine is comparatively small, usually not over one part in a thousand. Gently warm about an ounce of urine, pour it into a conical wine-glass, in which a few drops of muriatic acid have been previously placed. In twelve to 24 hours, very small brown crystals will be seen floating on the surface, resting on the sides, and settling to the bottom of the glass. By dexterous agitation these may all be collected at the bottom, as a mass of red sand, and the

† Dr. Bird.

supernatant liquid may be decanted, and the crystals may be washed by adding water, agitating, and allowing a little time for subsidence, when the water may be decanted. A drop of the water, containing these crystals, should be placed on a slip of glass, or in a watch-glass, and examined by the microscope. The acid will then be seen mostly in elongated spiculæ, or stars, and sometimes in square or rhombic (diamond-shaped) plates. This acid is the true "red sand" or gravel. Without a microscope, it can be distinguished by its reddish color, its hardness, insolubility, and by its weight, by which it settles so much like sand, and may be washed so perfectly clean. It is a little more satisfactory to see a larger quantity than is afforded by a wine-glass full of urine, and I have often prepared it in a Florence flask, two-thirds full of urine, by adding 30 drops of muriatic acid, mixing thoroughly by agitation, and allowing it to stand two or three days in cool weather, with occasional slight agitation towards the last, in order to collect the crystals at the bottom.

Birds and serpents discharge their urine and their feces by a common orifice, their urine being of a tolerably solid consistency, of a white color, and consisting of urate of ammonia. To procure uric or lithic acid, the white urine of a large serpent, the Boa Constrictor for example, is boiled with a solution of potassa until the urate of potassa is formed, which may afterwards be decomposed by adding muriatic acid, and thus precipitating the uric acid. This, it will be recollected, is the acid which is sometimes precipitated spontaneously in the kidneys or in the bladder, giving rise to what is popularly called "the gravel," a condition which calls for the administration of alkaline remedies, according to the above quoted passage from Dr. B. Jones.

10*th*. *Examination for Sugar*.—Diabetic urine has usually a high specific gravity, but this may be caused by an excess of urea. Several tests are given, in the standard works on the subject, for detecting sugar in urine. After repeating several of the processes given, I am better satisfied with a test, perhaps the most ancient, and certainly one of the most simple. I mean the fermentation test. Take a few ounces of the suspected urine, add a little yeast to it, and expose it at a temperature between 60 and 100 degrees Ft., in such a manner that if fermentation ensues, and carbonic is evolved, it will be collected. The most simple arrangement is, to fill a long four oz. vial with the urine and the yeast, and inverting it in a saucer, having already a little urine or water in it, support it uprightly, by any convenient means, and leave it exposed to the temperature already named, 60° to 100°. If sugar be present, in about three hours small bubbles will be seen accumulating about the yeast, and they will gradually escape and rise to what is now the upper end of the vial. Authorities say that there will be a cubic inch of carbonic acid gas evolved, for every grain of sugar in the urine. It is evident that in the above arrangement, with the inverted vial, as soon as the gas begins to accumulate, it displaces a portion of the urine into the saucer from which no gas can afterwards be collected. To obviate this, an apparatus may be adopted, consisting of a short bottle, to which a tight cork with a bent tube is adopted, the tube terminating openly under another bottle or small receiver, inverted with its mouth under water in the ordinary manner of collecting gases. In my own experiments on urine, to which a weighed quantity of grape sugar has been purposely added, although I have always procured carbonic acid, I have never obtained so much as the authorities have indicated.

*Apparatus required for the Clinical Examination of the Urine.*—In the course of Lectures of Chemistry in the Medical College of Ohio, for the session of 1852 and 3, I was desirous of putting into the hands of the Medical Students such instruments as would enable them to repeat most of the important experiments on urine and blood at their own rooms, where they would be compelled to perform their own manipulations, and thus become masters of the subject. After several trials, I devised the following instruments, which answers not only the purpose proposed, but may be used for a great variety of chemical experiments.

1st. A small furnace and water bath. For this purpose an apparatus already in the market, under the name of the Nurse lamp, with some new additions, answers well. It is commonly made of tin, and may be had for about 75 cents, including the cylinder, the spirit lamp, a steam bath, or water boiler, and a smaller tin vessel to use over the water bath. These are all made of tin, and will answer the purpose if carefully used, but are liable to be unsoldered if the boiler gets empty of water, or if the lamp wick gets too high, and the spirit in the lamp gets to boiling, and fills the whole affair with the flame of alcohol. This accident calls upon one to envelop the whole furnace in a woolen cloth, or indeed in any cloth which may be seized at the moment. From the liability to be unsoldered, a nurse lamp, made entirely of copper, brazed together, is preferable. Two pieces of additional apparatus sometimes furnished, are also convenient, a little tea kettle, about four inches in diameter, and a glass or earthen cup, to be used over the water bath. The dimensions of this nurse lamp, are about 8 inches high, and 4 inches in diameter, with a boiler 4 inches in diameter at top and $3\frac{1}{2}$ at bottom, $3\frac{3}{4}$ inches deep, having a shallower cup, the evaporator, $3\frac{1}{4}$ deep, to fit into it at the top, in order to hold things to be submitted to the water bath heat. The glass or earthen vessel should be similarly shaped, and is used in the same place. These vessels are furnished with a cover like that of a coffee pot, and may be used for making any kind of infusion requiring the heat of the water bath.

The spirit lamp should be two and three fourth inches in diameter, and two inches high, with a wick tube nearly one inch high above the lamp. The door of the cylinder for receiving the lamp should be three inches square. I have omitted to mention, that besides the lamp door in the cylinder, there should be two air holes, three-fourth inches in diameter, about one and three-fourth inches below the top of the cylinder.

2. Besides these things which are usually furnished with the nurse lamp, I have added several tin dishes four and one-fourth inches in diameter, and " raised" or rather depressed three-fourths of an inch, like a watch glass or saucer. Indeed each one is an inverted coffee-pot cover without its soldered rim. They are furnished at the tin furnishing stores at 20 cts per dozen. These are to be used as evaporators over the water bath, where glass of earthen evaporators may be substituted. A neat porcelain saucer becomes an elegant evaporating vessel over the boiler of the nurse lamp. Some of the above tin capsules, may have holes cut through them in the middle, from one to two inches in diameter, when they become holders for large watch crystals to be used over the boiler as evaporating vessels.

3. The Crystal Holder. This is made of Russian sheet iron, and consists of a circular part two and a half inches in diameter, and " raised" or dished by hammering one-fourth of an inch deep, and a handle like a spoon handle two and a half inches long, and about one inch wide, made of one and the same piece of iron. The object of this instrument is to be enabled to hold a watch crystal placed in it over a lamp or candle for boiling or evaporating. The watch crystal held naked over a lamp or candle is apt to be broken, but when held in this iron holder it is protected. The bowl or dish of the holder should be a little flatter than the crystal to be held, in order that the crystal may touch in the centre only, thus communicating the heat to the centre, where it is covered by the liquid, and not to the circumference, where it is dry and will become so hot, that when the liquid boils up upon it, the glass will be broken.

4. The urinometer.

5. The glass tube capable of receiving the urinometer when in use.

6. A small thermometer.

7. Several chemical re-agents as such:

Red Litmus paper;
Blue Litmus paper;
Muriatic acid;
Colorless nit. acid;
Caustic potassa.

8. A small accromatic microscope is desirable, but much can be done without it.

As was intimated at the commencement of this paper, it was not my intention to describe the diseases of which the abnormal conditions of the urine are the diagnostic symptoms. My purpose is answered, when I enable the physician to examine the urine in such a manner, as to ascertain with accuracy its characters, and to identify its deposits with scientific discrimination. The society are referred to the standard works on the practice of medicine, for the pathology and treatment which will be intelligible, *only* when the "clinical examinations" have been properly conducted. I have omitted any remarks on the detection of the common inorganic salts of the urine, such as the sulphates, muriates, phosphates. &c., of soda, potassa, lime, magnesia. These may be exhibited by the usual chemical tests. The whole mass of inorganic salts or the fixed salts of the urine may be obtained altogether by boiling down the urine to dryness, and urging the heat to redness, by which all the organic matters are charred. It is not necessary that the black spongy mass of charcoal should be burned out; for if the charring be complete the salts may be removed by filtering water through the pulverized mass. The clear lixivium yields by evaporation clean white crystaline salts.

Oxaluria is a disease or a symptom of disease so lately discovered, and has been so graphically described by Dr. Bird, that I am tempted to quote the following passage from his work. He says:

"Persons afflicted with the form of the disease referable to this class, are generally remarkably depressed in spirits, and their melancholy aspect has often led me to suspect the presence of oxalic acid in the urine. Sometimes a peculiar lurid greenish hue of the surface has been observed; but more generally the face has the dark and dingy aspect common in some forms of dyspepsia, in which the functions of the liver are deranged. They are generally much emaciated, except in slight cases, extremely nervous, and painfully susceptible of external impressions, often hypochondriacal to an extreme degree, and in very many cases labor under the impression that they are about to fall victims to consumption. They complain bitterly of an incapacity of exerting themselves, the slightest exertion bringing on fatigue. In temper they are irritable and excitable. In men, the sexual power is deficient, and often absent, an effect owing probably to the exhaustion produced by the excessive secretion of urea, so common in this affection. A severe and constant pain or sense of weight across the loins is generally a prominent symptom, with often some amount of iritability of the bladder. The mental faculties are but little affected, loss of memory being sometimes present. Well marked dyspeptic feelings are always complained of. Indeed in most of the cases in which I have been consulted, I have been generally told that the patient was ailing, loosing flesh, health and spirits daily; or remaining persistently ill and weak, without any definite or demonstrable cause. The urine is always of high spec. gravity, often within the diabetic range, and seldom below 1025 or 1030. This spec. grav. depending upon not only an excess of urea, for the urine generally crystalizes readily with nit. acid, but upon the existence of an abnormally large proportion of the extractive matters of the secretion. It is invariably acid, often excesssively so. The tendency to eruptions of minute furunculi, and even sometimes of large boils, is an exceedingly frequent concomitant of the state of urine under consideration, and a striking indication of the depressed state of the general health. In a few cases the patients have been suspected of being phthisical. It is, however, remarkable, that I have yet met with very few cases in which phthisis was present. In very few instances only have I seen the cases terminate in the formation of calculus."

Mental anxiety is mentioned as a cause of oxaluria accompanied by irritability of the bladder, and an increased quantity of urate of ammonia. Lawyers when suffering anxiety about their causes, and studying them intensely, are subject to the disease.

